# 25-Hydroxyvitamin D and Pre-Clinical Alterations in Inflammatory and Hemostatic Markers: A Cross Sectional Analysis in the 1958 British Birth Cohort

**DOI:** 10.1371/journal.pone.0010801

**Published:** 2010-05-24

**Authors:** Elina Hyppönen, Diane Berry, Mario Cortina-Borja, Chris Power

**Affiliations:** Medical Research Council Centre for Epidemiology of Child Health and Centre for Paediatric Epidemiology and Biostatistics, University College London Institute of Child Health, London, United Kingdom; Leiden University Medical Center, Netherlands

## Abstract

**Background:**

Vitamin D deficiency has been suggested as a cardiovascular risk factor, but little is known about underlying mechanisms or associations with inflammatory or hemostatic markers. Our aim was to investigate the association between 25-hydroxyvitamin D [25(OH)D, a measure for vitamin D status] concentrations with pre-clinical variations in markers of inflammation and hemostasis.

**Methodology/Principal Findings:**

Serum concentrations of 25(OH)D, C-reactive protein (CRP), fibrinogen, D-dimer, tissue plasminogen activator (tPA) antigen, and von Willebrand factor (vWF) were measured in a large population based study of British whites (aged 45y). Participants for the current investigation were restricted to individuals free of drug treated cardiovascular disease (n = 6538). Adjusted for sex and month, 25(OH)D was inversely associated with all outcomes (*p*≤0.015 for all), but associations with CRP, fibrinogen, and vWF were explained by adiposity. Association with tPA persisted after full adjustment (body mass index, waist circumference, physical activity, TV watching, smoking, alcohol consumption, social class, sex, and month), and average concentrations were 18.44% (95% CI 8.13, 28.75) lower for 25(OH)D ≥75 nmol/l compared to <25 nmol/l. D-dimer concentrations were lower for participants with 25(OH)D 50–90nmol/l compared to others (quadratic term *p* = 0.01). We also examined seasonal variation in hemostatic and inflammatory markers, and evaluated 25(OH)D contribution to the observed patterns using mediation models. TPA concentrations varied by season (*p* = 0.02), and much of this pattern was related to fluctuations in 25(OH)D concentrations (*p*≤0.001). Some evidence of a seasonal variation was observed also for fibrinogen, D-dimer and vWF (*p*<0.05 for all), with 25(OH)D mediating some of the pattern for fibrinogen and D-dimer, but not vWF.

**Conclusions:**

Current vitamin D status was associated with tPA concentrations, and to a lesser degree with fibrinogen and D-dimer, suggesting that vitamin D status/intake may be important for maintaining antithrombotic homeostasis.

## Introduction

Vitamin D deficiency has been suggested to contribute to the high and rising worldwide prevalence of cardiovascular disease (CVD) [1]. Vitamin D is a hormone precursor, which before exerting its metabolic effects undergoes two successive hydroxylations. The first hydroxylation converts vitamin D to 25-hydroxyvitamin D [25(OH)D, which provides an indicator for nutritional vitamin D status] [2] and the second to the main active hormonal form, 1,25-dihydroxyvitamin D [1,25(OH)_2_D]. Hormonal vitamin D activity is found throughout human circulatory tissue and 1,25(OH)_2_D production has been demonstrated in endothelial cells of blood vessels [3] . Vitamin D receptors (VDR, mediating the genomic hormonal actions) are expressed in endothelial cells, cardiomyocytes and vascular smooth muscle cells, including those in the coronary arteries [4], [5]. VDR knockout mice show signs of enhanced thrombogenicity [6].

The strongest evidence for a relation between vitamin D metabolism and CVD risk has been obtained from clinical studies reporting a marked reduction in mortality following administration of vitamin D analogues to patients with end-stage renal disease [7], [8], [9]. Evidence for an association between vitamin D status and subsequent risk of CVD was found in recent prospective studies on myocardial infarction [10] and cardiovascular mortality [11] ; both of these studies reported a two-fold increase in the risk of CVD for vitamin D insufficient participants compared to others. Concentration of the active hormone has been related to the degree of arterial calcification in individuals at increased risk of myocardial infarction [12] and an inverse association has been reported between serum 25(OH)D concentration with carotid artery intima-media thickness [13], myocardial infarction [14], metabolic syndrome [15], [16], and CVD [17], [18].

To date there is relatively little evidence from population-based studies on the associations of 25(OH)D with indicators of inflammation or hemostasis, and to what extent possible co-variation is affected by adiposity. Obesity is a key determinant for the circulating 25(OH)D concentrations [19] and also an important cardiovascular risk factor. Consequently, it is difficult to separate the effects of vitamin D status and adiposity when evaluating their influences on cardiovascular risk. In order to obtain further evidence for a possible independent contribution of current vitamin D status on pre-clinical alterations in markers of inflammation and hemostasis, we evaluated seasonal patterns in inflammatory and hemostatic markers and the strength of the effect mediation by 25(OH)D. This approach is likely to be informative, as due to the strong influence of sun induced skin synthesis, 25(OH)D concentrations vary greatly by season [19], while little variation would be expected for adiposity. Our aim was to investigate the association between 25(OH)D, adiposity (body mass index, waist circumference) and pre-clinical variations in the available risk markers (namely CRP, fibrinogen, D-dimer, tPA, and von Willebrand factor). In these analyses, we used information from the nationwide 1958 British birth cohort (1958BC) on over 6500 middle aged participants. We hypothesized that if vitamin D intake affects the markers under investigation then further evidence for an association should be obtained through analysing the contribution of 25(OH)D to the seasonal variation in markers of inflammation and hemostasis.

## Results

**Figure 1:**
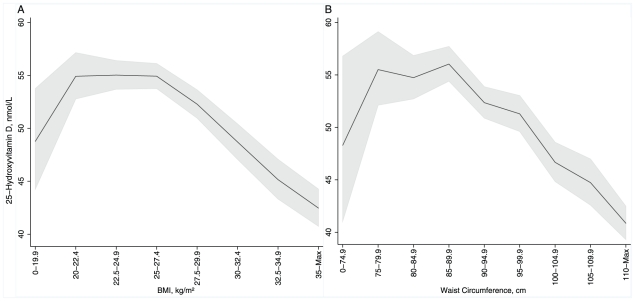
Variation in the average 25(OH)D concentration by body mass index (A) and waist circumference (B). Values are geometric means (95% confidence intervals) standardized by sex.

The geometric mean of 25(OH)D concentration was 52.77 nmol/l (95% CI 52.18, 53.36). Table 1 shows the distribution of 25(OH)D concentrations by social and lifestyle characteristics. For both BMI and waist circumference (the available adiposity indicators) the association with 25(OH)D was non-linear (LRT curvature p≤0.0001 and p = 0.04, respectively), with the highest 25-hydoxyvitamin D concentrations observed for individuals with normal weight (Figure 1). There was a steep decline in the average 25(OH)D concentration by increasing adiposity and a smaller reduction for the very lean.

**Table 1:** Distribution of 25-hydroxyvitamin D concentration by background, lifestyle and social characteristics in the 1958 British birth cohort.

		25-hydroxyvitamin D, nmol/l		
	Number	Geometric Mean*	<25 nmol/l	>125 nmol/l
	(%)	(95%CI)	%* (*n*)	%* (*n*)
**Sex**				
Men	3270 (50.0)	53.6 (52.8, 54.5)	6.2 (203)	1.4 (46)
Women	3268 (50.0)	51.9 (51.1, 52.8)	8.4 (273)	1.4 (45)
*p-value*		0.003	0.0007	0.9
**Body Mass index**				
<25	2361 (36.1)	55.1 (54.0, 56.2)	7.5 (178)	2.2 (52)
25–30	2737 (41.9)	54.2 (53.3, 55.1)	5.8 (159)	1.2 (32)
>30	1440 (22.0)	46.8 (45.7, 47.9)	9.7 (139)	0.5 (7)
*p-value*		≤0.0001	0.04	≤0.0001
**Waist circumference** **				
Quartile 1	1642 (25.1)	57.4 (56.0, 58.7)	6.8 (111)	2.8 (46)
Quartile 2	1639 (25.1)	55.2 (54.0, 56.4)	6.2 (102)	1.8 (29)
Quartile 3	1627 (24.9)	52.6 (51.4, 53.7)	6.1 (100)	0.7 (11)
Quartile 4	1618 (24.8)	46.6 (45.6, 47.6)	9.9 (160)	0.2 (4)
Unknown	0.2 (12)	44.8 (29.4, 68.3)	25.0 (3)	8.3 (1)
*p-value*		≤0.0001	0.003	≤0.0001
**Vigorous activity**				
No	3206 (49.0)	49.6 (48.8, 50.4)	9.4 (300)	1.2 (37)
Yes	3236 (49.5)	56.3 (55.4, 57.2)	5.0 (163)	1.7 (54)
Unknown	96 (1.5)	48.0 (43.2, 53.2)	13.5 (13)	0.0 (0)
*p-value*		≤0.0001	≤0.0001	0.07
**TV watching/use of PC**				
<1 hours/day	745 (11.4)	56.3 (54.5, 58.2)	5.8 (43)	2.3 (17)
1–2 hours/day	3455 (52.8)	54.9 (54.1, 55.7)	5.5 (191)	1.6 (54)
≥3 hours/day	2056 (31.4)	48.9 (47.9, 49.9)	9.9 (204)	0.9 (19)
Unknown	282 (4.3)	47.7 (44.9, 50.5)	13.5 (38)	0.4 (1)
*p-value*		≤0.0001	≤0.0001	0.01
**Smoking**				
None	3039 (46.5)	54.2 (53.3, 55.1)	6.1 (185)	1.3 (41)
Ex-smoker	1795 (27.5)	54.8 (53.7, 55.9)	5.1 (91)	1.3 (23)
1–19 per day	762 (11.7)	50.3 (48.5, 52.1)	10.4 (79)	1.7 (13)
≥20 per day	720 (11.0)	45.5 (43.8, 47.2)	14.4 (104)	1.9 (14)
Unknown	222 (3.4)	51.8 (48.8, 55.1)	7.7 (17)	0.0 (0)
*p-value*		≤0.0001	≤0.0001	0.2
**Alcohol consumption**				
Non-drinker	378 (5.8)	46.1 (44.0, 48.4)	11.4 (43)	0.0 (0)
Light <7 drinks/wk	3155 (48.3)	52.1 (51.3, 52.9)	7.5 (237)	1.0 (31)
Moderate 7–13 drinks/wk	1651 (25.3)	55.6 (54.4, 56.9)	5.6 (92)	1.8 (30)
Heavy 14–21 drinks/wk	746 (11.4)	55.9 (54.1, 57.8)	4.8 (36)	2.5 (19)
Very heavy >21 drinks/wk	590 (9.0)	49.7 (47.7, 51.8)	10.7 (63)	1.7 (10)
Unknown	18 ( 0.3)	41.5 (31.5, 54.6)	27.8 (5)	5.6 (1)
*p-value*		0.01	0.9	0.0003
**Adult social class (2000)** †				
I & II	2675 (40.9)	53.3 (52.4, 54.2)	6.7 (180)	1.4 (38)
III non-manual	1363 (20.9)	52.2 (50.9, 53.5)	7.9 (107)	1.1 (15)
III manual	1220 (18.7)	54.5 (53.1, 56.0)	6.1 (74)	2.0 (25)
IV & IV	1013 (15.5)	51.2 (49.8, 52.7)	8.0 (81)	1.0 (10)
Other	267 (4.1)	48.8 (45.9, 51.8)	12.7 (34)	1.1 (3)
*p-value*		0.007	0.02	0.8

*Values are *n* (%) or geometric mean. *p*-values from test for trend in linear or logistic regression adjusted for season and sex. Unknown values excluded.

**Waist circumference quartiles: for men; 65.4–90.6, 90.7–96.7, 96.8–103.5, 103.6–151.2 cm; for women; 56.2–75.8, 75.9–82.6, 82.7–91.6, 91.7–138.3 cm.

†Classes I&II are managerial/professional, IV/V unskilled manual. “Other” includes cohort members who are institutionalised, retired, unemployed and other unclassifiable.

**Figure 2:**
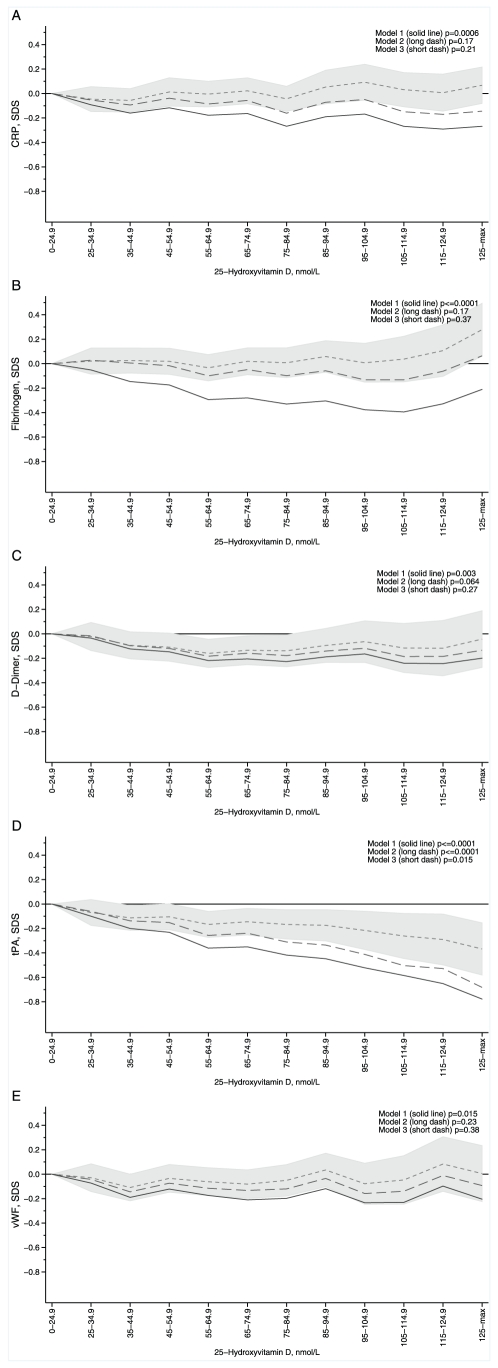
Variation in C-reactive protein (A), fibrinogen (B), D-dimer (C), tissue plasminogen activator (D), and von Willebrand factor (E) by 25(OH)D concentration. Model 1 (solid line): adjusted for month of measurement and sex. Model 2 (dashed, short): adjusted for lifestyle and social indicators (physical activity, time spent watching TV/using PC, smoking, alcohol consumption and birth and adult social class), month of measurement and sex. Model 3 (dashed, long): adjusted for adiposity (BMI and waist circumference), lifestyle/social indicators, month of measurement, and sex. Values are coefficients from linear regression (reference <25nmol/l), 95% confidence intervals presented for Model 3 by the shaded area.

Adjusted for sex and month of measurement only, 25(OH)D was associated with all inflammatory and hemostatic outcomes (p≤0.01 for CRP, fibrinogen, D-dimer and tPA, p = 0.015 for vWF, Figure 2). Associations between 25(OH)D with CRP, fibrinogen, and vWF were strongly attenuated after adjustment for lifestyle and social indicators, and no evidence for an independent inverse association remained after further adjustment for adiposity. For fibrinogen, after full adjustment for adiposity, lifestyle and social indicators, there was a curved association with some suggestion for increased levels at 25(OH)D concentrations of ≥125 nmol/l (LRT quadratic term p = 0.06 Figure 2B). 25(OH)D had a curved association with D-dimer, and after full adjustment participants with 25(OH)D between 50–90 nmol/l tended to have lower levels (LRT quadratic term p = 0.01 Figure 2C). The association between 25(OH)D and tPA was not strongly affected by adjustment for lifestyle factors, and a significant inverse trend was apparent after further adjustment for adiposity although the effect size was halved (Figure 2D). After full adjustment, participants with 25(OH)D ≥75 nmol/l had on average 18.44% (95% CI 8.13, 28.75) lower tPA concentrations compared to those with <25 nmol/l. There was no evidence for effect modification by obesity on the association between 25(OH)D and the inflammatory or hemostatic outcomes (p>0.13 for all comparisons).

**Figure 3:**
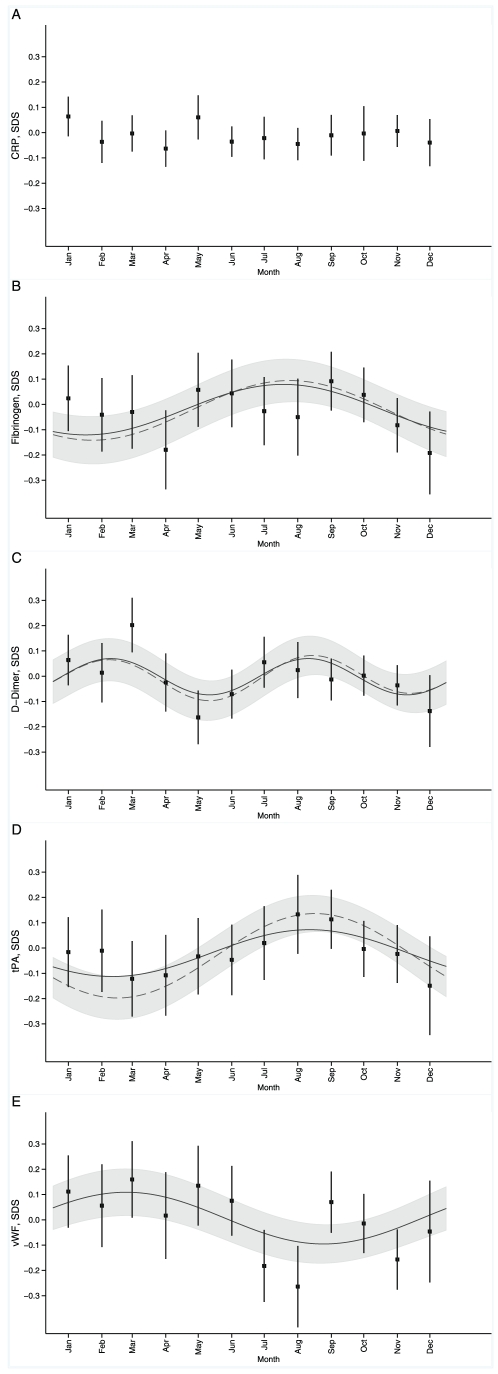
Seasonal variation in C-reactive protein (A), fibrinogen (B), D-dimer (C), tissue plasminogen activator (D), and von Willebrand factor (E). Values are from the partial regression of the harmonic components; Model 1 (solid line) adjusted for respiratory infections, alcohol consumption, PC/TV time, physical activity and social class at birth and adulthood, and Model 2 (dashed line, shown with 95% confidence intervals) in addition to above adjusted for 25-hydroxyvitamin D. Tick marks denote average concentrations (SDS, predicted from random effects models) with 95% confidence intervals shown by error bars. Predicted means for CRP from linear models, no seasonal pattern observed (p>0.8). *p-values from the product of coefficient mediation test used to assess the 25(OH)D mediation effect on the seasonal patterns in the outcomes.

Given the strong influence of season on 25(OH)D concentrations [19], we evaluated seasonal variation in hemostatic and inflammatory markers, and tested mediation effects of 25(OH)D in the observed patterns. Fibrinogen, tPA, D-dimer, and vWF but not CRP had significant seasonal patterns (*p* = 0.03, *p* = 0.02, *p* = 0.02, *p* = 0.01 and *p* = 0.8, respectively, Figure 3). The strongest effect mediation by 25(OH)D was seen in the pattern of tPA (*p*<0.001), with 25(OH)D contributing to a lesser extent to seasonal variation in D-dimer and fibrinogen. The seasonal variation seen in vWF was not affected by 25(OH)D (*p* = 0.99).

## Discussion

We observed a strong cross-sectional association between circulating 25(OH)D and tPA concentrations in participants free of clinical CVD, and a seasonal pattern for tPA that was largely mediated by 25(OH)D in this population. These findings, together with the weaker evidence observed for a relation of 25(OH)D with D-dimer and fibrinogen, suggest a role for current vitamin D status in determining thrombolytic profile before progression to CVD.

A specific methodological challenge for these cross-sectional analyses arose from the strong association of adiposity both with 25(OH)D concentrations and the inflammatory/hemostatic markers under study. In addition to the conventional approach of evaluating the direct association between 25(OH)D and the outcomes adjusting for potential confounders (most importantly, body mass index and waist circumference), we evaluated seasonal variation in the outcomes and the mediating influence of 25(OH)D on the observed patterns. These analyses supported a relation of 25(OH)D with tPA, and interestingly, also to lesser extent with D-dimer and fibrinogen. The seasonal pattern seen in vWF was not affected by 25(OH)D, nor did we observe evidence for a direct cross-sectional association, hence, this confirms the lack of evidence for any association between vitamin D status and circulating vWF concentrations in our study.

### Comparison with other studies

Risk of myocardial infarction and other thrombotic complications is typically higher during the winter months than during the summer [20], [21], and in line with our study, fibrinogen (but not CRP) has been reported to vary by season [22]. However, there is little information on the direct associations between 25(OH)D and circulating markers of hemostasis in apparently healthy adults. In line with our findings, an earlier smaller study found the association between 25(OH)D and CRP to be explained by adiposity, while an independent relation persisted between 25(OH)D and tPA [23]. The lack of evidence for an association between current vitamin D status and CRP agrees with recent observations in other populations [24], including intervention studies where vitamin D supplementation at varying dosages (700–3332 IU per day) have failed to achieve changes in CRP concentrations [25], [26], [27]. There is, however, one earlier intervention which showed a significant (22%) fall in CRP following vitamin D supplementation (50,000IU every three months over one year) in vitamin D deficient British Bangladeshi adults [28]. It is possible that associations between 25(OH)D and CRP might not be detected in general population studies if effects are confined to extreme vitamin D deficient groups.

### Explanations

Increased concentrations of tPA and D-dimer are thought to serve as markers for aggravated fibrinolytic activity reflecting increased future burden of CVD [29]. Hence, the inverse associations of 25(OH)D with tPA and D-dimer observed in our study support the role of vitamin D metabolism in maintaining antithrombotic homeostasis. The direct influences of hormonal vitamin D axis on hemostasis are not well established, although recent gene-expression studies suggest that vitamin D analogues may suppress thrombogenicity and enhance fibrinolysis thereby reducing intimal plaque formation [30], [31]. Vitamin D analogues have been observed to suppress PAI-1 expression in human coronary artery smooth muscle cells [32]. Up regulation of PAI-1 has been associated with increased risk of CVD, and it has been suggested that suppression of PAI-1 expression may contribute to the observations on improved survival among patients with chronic kidney disease who are taking vitamin D analogues [7], [32].

Hypovitaminosis D is believed to have wide-ranging influences on vascular physiology, which include both direct (e.g. influences on endothelial cells) and indirect pathways (endocrine, immunomodulatory) [33]. Vitamin D toxicity has been associated with adverse effects on vascular calcification, but available data indicates that calcification is increased also in hypovitaminosis D [34], [35]. Associations with increased blood pressure are believed to be mediated through decreased renin production [36] and it has been suggested that (independently of blood pressure), this could also affect vascular stiffness [33]. Vitamin D might also exert anti-proliferative effects on vascular smooth muscle cells, thereby affecting myocardial cell hypertrophy and proliferation.

Discussion regarding optimal status for 25(OH)D concentration is ongoing, and there is some debate about whether a threshold exists [37], [38], [39], [40]. The curved association between 25(OH)D and D-dimer, together with the suggestive elevations in fibrinogen and CRP at the extreme of high concentrations observed in this study, could support a threshold effect with the optimal concentration being between 60 and 120nmol/l. These results are in line with an evaluation using data on multiple health outcomes (including bone mineral density, lower extremity function, risk of falls, fractures and colorectal cancer) [40], which corresponds to an earlier consensus statement on osteoporosis [37] suggesting 75nmol/l as the lower reference value. Earlier studies have provided tentative evidence for a trend towards lower bone mineral density and prolonged sit-to-stand time at a higher extreme of 25(OH)D concentrations (>140 nmol/l) [40].

### Methodological considerations

The main strength of this study lies in the large sample of participants, which provided adequate power for detailed investigation of the associations between these inter-related health indicators. Moreover, as data collection covered the full seasonal range, we were able to obtain further support for the key findings from the independent evaluation of seasonal patterns in inflammatory and hemostatic markers. Given the exceptional information available from the 1958BC, we were able to adjust for multiple factors in our analyses thereby controlling for confounding introduced by demographic, lifestyle or social variations. Final models evaluating the independent effect of 25(OH)D on inflammatory and hemostatic outcomes were adjusted for quadratic terms in both BMI and waist circumference in order to control for adiposity as fully as possible. The full attenuation of the association between 25(OH)D with CRP and fibrinogen after adjustment for the available indicators suggests that these measures were sufficient for this purpose.

Comparison between the effect of adjustment for 25(OH)D concentrations in the observed seasonal patterns in the inflammatory/hemostatic factors, and the direct associations between 25(OH)D and these outcomes, demonstrates the limitations of cross-sectional analysis of data and the problem of possible over/under adjustment. Given the strong influence of obesity on 25(OH)D concentrations, the latter would be expected to be associated with any factor that is strongly related to obesity (given a tolerable degree of measurement error and sufficient sample size). This argues for the need to adjust for obesity fully to reduce the likelihood of a false positive association due to confounding. However, it could also be argued that adjustment for adiposity may lead to an underestimation of associations between 25(OH)D and inflammatory/hemostatic markers given that adiposity is a key determinant for 25(OH)D [19]. Possible over-adjustment could explain why we observed some evidence for effect mediation by 25(OH)D on our seasonal modeling of fibrinogen, while the inverse relation seen in the unadjusted cross-sectional analyses between these two factors was fully attenuated by the adjustment for indicators of adiposity and lifestyle/social class.

Some further limitations need to be considered in relation to these findings. Given the observational design, we cannot prove causality or fully discount residual confounding by unmeasured variations. Residual confounding may also affect our seasonal mediation models; however, relevant confounders are likely to differ given that potential factors presumed important for the direct associations (such as adiposity) would not necessarily have seasonal patterns. Although 25(OH)D is the best indicator for vitamin D status in humans [2] information on serum parathyroid levels or 1,25(OH)_2_D is not available and this precludes more detailed investigation of vitamin D metabolism. Furthermore, information was available only for the most commonly used CVD risk markers, while other, perhaps more relevant, indicators were not measured. Finally, although the 1958BC has been reported to remain generally representative of the current UK population, there is an underestimation of some minority groups [41]. These analyses were restricted to individuals of white Caucasian origin, which will reduce population stratification, however, generalization of these findings to other ethnic groups should be done with caution.

### Conclusions

Current vitamin D status was associated with circulating concentrations of tPA and D-dimer, which may suggest a role for vitamin D in maintaining antithrombotic homeostasis. Further studies, including randomised controlled trials, are needed to demonstrate the role of vitamin D metabolism in cardiovascular health, and whether vitamin D supplementation or improved vitamin D status could have beneficial effects.

## Methods

Written consent for the use of information in medical studies was obtained from the cohort members. The 45y biomedical survey of the 1958BC was approved by the South-East Multi-Centre Research Ethics Committee (ref: 01/1/44).

Participants in this study are from the 1958 British birth cohort, which included all births in England, Scotland, and Wales during one week in March 1958 (*n* = 17,416) [41], [42]. Between September 2002 and April 2004 a target population of 11,971 individuals currently living in Britain were contacted aged 44y (31.1%) to 46y (0.4%): 78% (*n* = 9377) participated in the biomedical survey and 7591 (80%) also provided blood samples from which 25(OH)D was measured [19], [42]. This sample is representative of the surviving cohort; however, as we have reported previously, there is some under-representation of specific minority groups [41]. The 1958BC is largely a white European population (98%); for these analyses 154 individuals of other ethnicity groups were excluded. We further excluded one participant who was pregnant at the time of survey. As the main focus of the analyses was to evaluate the association of 25(OH)D concentrations on pre-clinical alterations in inflammatory and hemostatic markers, we excluded all participants (*n* = 532) who used any type of medication used to treat cardiovascular problems (BNF code 2: Cardiovascular systems). We further excluded participants with missing data on BMI or inflammatory/hemostatic markers (n = 366) leaving 6538 individuals for the main analyses.

### Laboratory analyses

Venous blood samples were obtained without prior fasting and posted to collaborating laboratories. Fibrinogen was determined by the Clauss method and CRP assayed by nephelometry (Dade Behring) on citrated plasma samples after one thaw cycle. vWF antigen was measured by Decollates enzyme-linked immunosorbent assay (elisa) and tPA antigen by Biopool elisa. 25(OH)D was measured using automated application of the IDS OCTEIA elisa on the Dade-Behring BEP2000 analyser (sensitivity of 5.0 nmol/l, linearity ≤155 nmol/l, intra-assay variation CV 5.3–7.4% and inter-assay variation CV 7.7–8.5%) [41], [43]. Heterogeneity of 25(OH)D concentrations measured by different assays is well-known, therefore levels were standardized according to the mean of the Vitamin D External Quality Assessment Scheme (DEQAS) [43].

Demographic, lifestyle and social factors have been described in detail previously [19], [42], [44], [45], [46]. In brief, weight, height and waist circumference were measured at 45y. Socio-economic position at birth (1958) and at age 42y was assessed using the Registrar General's occupational classification categorised as I &II (professional/managerial), III non-manual, III manual, IV & V (unskilled manual) [44]. Recreation Metabolic Equivalent of Task (MET) hours per week at 45y was derived from reported frequencies and usual durations for up to 37 activities, and published MET scores. We divided recreational activity into gender-specific quartiles. An additional category was created for implausibly high values (participants with weekly recreation hours of 3 standard deviations (SD) above the gender mean). Participants engaging in vigorous activity were those who recorded an activity with a MET score greater than six. Time spent watching a television or using a computer was reported at age 45y [19]. Information on smoking was based on smoking history recorded at ages 23y, 33y and 42y [46] and alcohol consumption on report from age 45y.

### Statistical Analysis

To describe the distribution of 25(OH)D concentration we used dichotomous indicators for levels below 25 nmol/l and above 125 nmol/l and a categorised factor into 25 nmol/l divisions with minimum <25 nmol/l and maximum ≥125 nmol/l tails. The natural logarithmic transformation was used to calculate geometric means to adjust the skewed distribution.

Inflammatory and hemostatic markers were transformed to gender-specific standard deviation scores (SDS) to compare variation across models. The SDS values were used as response variables in linear mixed effects regression models. Initial analyses included validation and graphical examination of data, statistical evaluation of linear and quadratic terms for 25(OH)D, adiposity measures (BMI and waist circumference), and single and joint effects of these measures on the inflammatory/hemostatic outcomes. Continuous measures were used in testing for interactions between the adiposity measures and 25(OH)D on the outcomes. For D-dimer three outlying observations were identified from graphical examination and model diagnostics (leverage and/or influence >2SD), and excluded from further analysis. We fitted linear regression models in three stages, starting with simple associations between 25(OH)D (categorized into 10 nmol/l groups, minimum <25 nmol/l and maximum ≥125 nmol/l tails) and the SDS inflammatory/hemostatic outcomes (model 1), next adjusting in addition for demographic, lifestyle and social factors (model 2), and finally adjusting for adiposity in addition to lifestyle and social factors (model 3). Models (1–3) included covariates gender and month of measurement and models with fibrinogen SDS as the outcome included laboratory assay batch. We also created an additional 25(OH)D category variable of <25, 25–74.9 and ≥75 nmol/l to summarize the effect size and repeated analyses for model 3. Missing information on the lifestyle factors (*n* = 514 with missing information on one or more factors) was imputed using the multiple imputation chained equations [47]. The models (1–3) were run on 10 imputed datasets and repeated for the sample restricted to participants with complete data on all confounders. The results were similar with both approaches; hence, results are only presented from the imputed models.

Seasonal variations were modeled using sine and cosine functions [48] with laboratory assay batch as a random effect on the intercept where appropriate (Likelihood Ratio Test (LRT) on between-batch variation *p*<0.05). The model equation was:
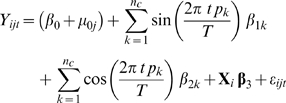
Where 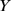 is the response variable, *i* denotes the individual within the assay batch *j* , *T* is the time period (*T* = 365 days), *n_c_* is the number of seasonal patterns with sequence (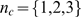) or combination of them, *p_k_* is the period for the season (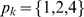) where 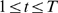, **X** *_i_*_3_ is a matrix of linear predictors for the vector of parameters **β**_3_ (inclusive of the mediator variable and seasonal confounders). The random effect in the intercept, 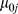, is defined by the laboratory assay batch as the dates of the blood samples were associated with the assay batches and we did not wish to estimate the effect of batch on the response; we assume 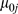
∼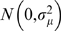. Finally, the observational error terms 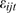 were assumed to be normally distributed with mean 0 and variance 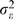.

Without the hierarchical structure implied by the random effects terms we may have under-estimated the standard error on the intercept. The *n_c_* seasonal patterns investigated were yearly, half yearly, quarterly, and all their combinations of were included if appropriate. Models were adjusted for the potential seasonal confounders of respiratory infections, alcohol consumption, PC/TV time, physical activity and social class at birth and adulthood. These resulting models were further adjusted for 25(OH)D, its dominant yearly pattern and then re-tested for subsidiary cycles.

To quantify the seasonal effect of 25(OH)D on the outcomes we used the concept of mediation analysis [47], [49] where season, as modeled with the sine/cosine transformation, was the independent variable and 25(OH)D acted as its mediator to the outcomes. The product of coefficients test [50] used in mediation analysis was extended to allow for the amplitude (the seasonal variation around the mean) as derived from the sine/cosine transformation. The final seasonal models predict the mean levels of the outcomes from the partial regression coefficients of the seasonal functions. All analyses were carried out using STATA, version 10.0 (StataCorp LP, College Station, TX).
